# Creating a Defined Process to Improve the Timeliness of Serious Safety Event Determination and Root Cause Analysis

**DOI:** 10.1097/pq9.0000000000000200

**Published:** 2019-08-07

**Authors:** Lane F. Donnelly, Tua Palangyo, Jessey Bargmann-Losche, Kiley Rogers, Mathew Wood, Andrew Y. Shin

**Affiliations:** From the * Center for Pediatric and Maternal Value, Lucile Packard Children’s Hospital – Stanford, Stanford Children’s Health; † Stanford University, School of Medicine, Palo Alto, CA, USA

## Abstract

Serious Safety Events (SSEs) are defined as events in which there is a deviation from clinically accepted performance standards, causation, and significant patient harm or death. Given the nature of SSEs, it is important that the processes for declaration of SSEs, the performance of a root cause analysis (RCA), and action plan formation occur quickly, such that the window for potential recurrence of similar events is as small as possible. This manuscript describes a process put in place to improve the timeliness of SSE determination and RCA conduction and evaluates the effect of the process change on these parameters.

**Methods:**

A causal analysis was performed of the baseline process to determine factors contributing to long process times. A new process was created and implemented both for the SSE determination process and the RCA completion process. We calculated the mean time for the pre-implementation phase (April 2016–December 2017) and the post-implementation phase (March 2018–January 2019) for both SSE determination and RCA completion. We evaluated differences with a two-sided *t* test assuming unequal variances.

**Results:**

Comparing pre- versus post- implementation phases, the mean time for SSE determination for events that met the SSE criteria decreased from 38.4 to 4.8 days (*P* < 0.0001), determination for events that did not meet the SSE criteria decreased from 38.4 to 3.8 days (*P* < 0.0001), and RCA completion time dropped from 118.0 to 26.2 days (*P* < 0.0001).

**Conclusions:**

A targeted intervention can significantly reduce SSE determination and RCA conduction times.

## INTRODUCTION

Serious Safety Events (SSEs) are defined as events in which there is a deviation from best practice care, causation, and significant patient harm or death.^1–4^ Over the past 10–15 years, SSE reduction has been a goal chosen by the community of children’s hospitals. Programs to successfully and significantly reduce SSEs were initially introduced at individual children’s hospitals.^2–4^ These programs were then spread to and successfully reduced SSEs within a network of hospitals, initially with six hospitals in the Ohio Children’s Collaborative^5^ and then within Solutions for Patient Safety (SPS)—a collaborative of now over 140 children’s hospitals.^6,7^

Given that SSEs are associated with deviation from best practice care and result in significant patient harm, identifying an event as an SSE, as well as analyzing the event and implementing an effective action plan to prevent recurrence is paramount. The timeliness of SSE determination and execution of a Root Cause Analysis (RCA) determine the length of time that a preventive action plan is not in place and the window of vulnerability for an SSE recurrence. Therefore, this timeframe should be as short as possible without compromising the quality of the processes. We describe a defined operational process put in place both for SSE determination and for RCA execution leveraging targeted timelines to promote a shared mental model. We compare the mean time from event discovery to SSE determination as well as the mean time to complete an RCA before and after the implementation of the newly designed processes for both tasks.

## METHODS

Our Institutional Review Board considered this project a quality improvement and not human subjects research. Therefore, it did not require review and approval. This improvement project was carried out at the Lucile Packard Children’s Hospital Stanford. The system operates ~400 licensed beds in a free-standing children’s and maternity hospital, associated ambulatory services, and is associated with Stanford University.

We calculated the mean time for the pre-implementation phase and the post-implementation phase for both SSE determination and RCA completion. The baseline period or pre-implementation phase was April 2016–December 2017. Aspects of the new process were rolled out during the implementation phase of January 2018–February 2018. The post-implementation phase was March 2018–January 2019.

### SSE Determination Process—Time Reduction

The definitions of SSEs and flow algorithms used to determine SSEs at this institution are based on those published by HPI^1^ and followed by SPS.

The quality and safety leadership at our institution changed. The new leadership perceived, based on their experiences at other institutions, that the length of time from discovery of a potential SSE to the declaration as an SSE or not an SSE was too long. A goal was set to reduce the SSE determination time to <7 days, chosen by consensus of the quality and safety team as it seemed realistic and could be achieved by having a weekly Event Review Meeting to discuss all potential SSEs.

#### Baseline SSE Determination Process.

The baseline SSE determination process was not well-defined. Chart review by the patient safety advisors would take place during days after event discovery. Safety advisors have a nursing background. They manage the incident reporting system, cull incident reports for cases that may be SSEs or precursor events, preparing such identified cases for review, and managing the RCA process. Safety advisors were in place both during the baseline and new SSE and RCA processes. Patient safety advisors would send SSE investigation notification emails to local and organizational leaders, identify individuals to be interviewed, and send interview request emails to the identified people and their direct supervisor. If there was no response to the interview request email, the advisors sent a second email and identified other individuals as potential interviewees. Events involving physician practice were referred to a specialty-specific Professional Practice Evaluation Committee (PPEC) for peer review. As the PPECs only meet monthly, getting information back to the patient safety team could take several months. The patient safety advisors would summarize the findings and present to the Patient Safety Oversight Committee for review and SSE determination. Patient Safety Oversight Committee occurred every 2 weeks and included hospital executives such as the chief executive officer, chief medical officer, chief nursing officer, and others.

#### Causal Analysis for Factors Contributing to Lengthy SSE Determination.

**Fig. 1.**
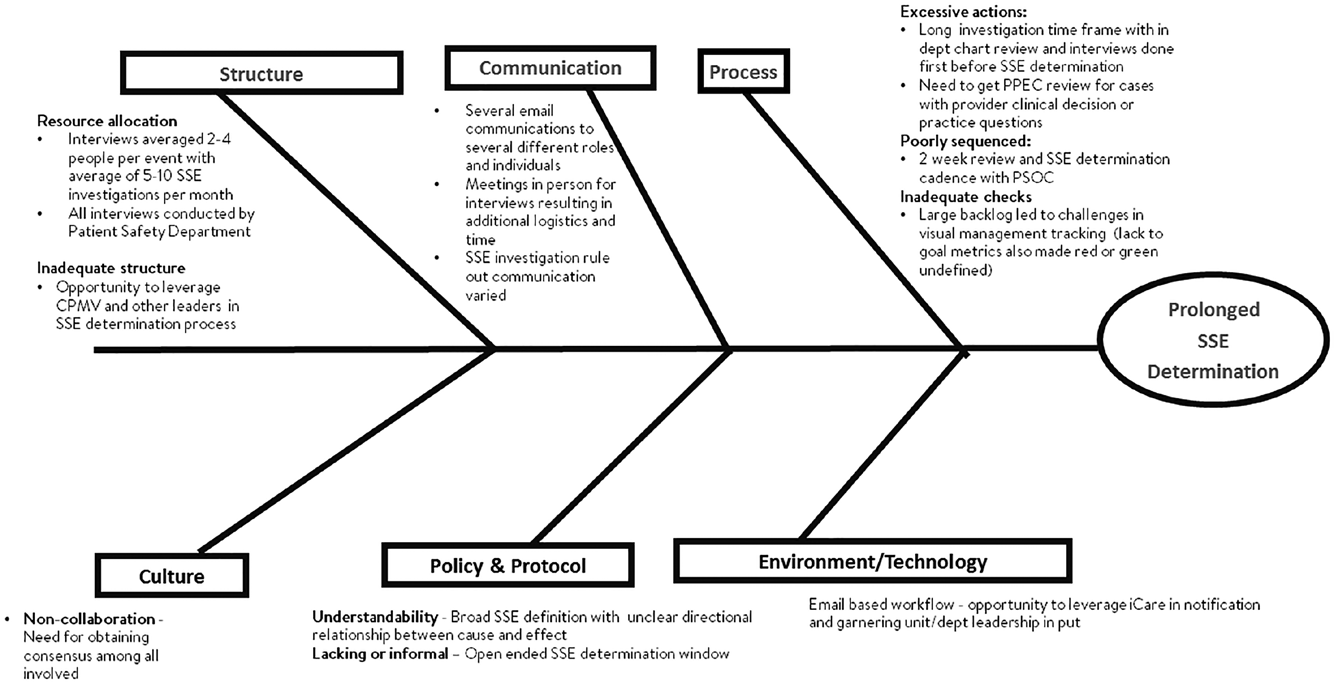
Cause and effect (Fishbone) diagram outlines factors which contributed to the long SSE determination times.

A causal analysis was performed to determine contributing factors to long determination times (Fig. 1). Dominant contributing factors included process issues related to the sequence of meetings and a cultural need for consensus building among hospital leadership and the leadership of the teams involved in an event, particularly among the physician groups.

#### New SSE Determination Process.

For the new process, when a potential SSE becomes known, the patient safety advisors perform chart review and perform any indicated phone interviews as soon as possible. We established an organizational expectation for patient care leadership teams and their role in the SSE determination process: a review of all safety events within 3 days of submission and immediate review of any event identified as a potential SSE. If they perceive events as high severity or high risk, the chief quality officer and other quality and safety leaders are immediately notified to facilitate direct risk mitigation. For events where physician practice is in question, the chief quality officer or other physicians from the quality and safety team are brought in to conduct those interviews.

We review potential SSEs at a weekly Event Review Meeting. The cases are prepared and presented by the patient safety advisors to the patient safety team, director of patient safety, chief quality officer, associate chief quality officers, director of the Center for Pediatric and Maternal Value, chief nursing officer, chief medical officer, and physician representatives with expertise in intensive care, obstetrics, and surgery. Case review summaries are provided and projected during the meeting. Attendees can call in from remote locations and follow the screen presentation online.

At the Event Review Meeting, there are two tasks for each case evaluated: (1) SSE determination (yes/no) and (2) the determination as to what type of further investigation is needed, if any. Potential investigations include full RCA, a process review (an analysis less intense than a full RCA), referral to the professional practice evaluation committee (PPEC), or some combination of the above. Note that the decisions concerning the two tasks mentioned above are independent. While most events determined to be an SSE undergo an RCA, due to the nature of the event, not all would benefit from the RCA process. Likewise, some events not deemed to be an SSE still undergo an RCA to address any significant systems issues.

SSE determination has three potential outcomes at the meeting: (1) The case does not meet the SSE criteria. (2) The case meets SSE criteria and determined to be an SSE. (3) Indeterminate case due to lack of information or lack of subspecialty expertise present at the Event Review Meeting. For indeterminate cases, we assign ownership of the case to a physician leader from the event review group, and we contact the appropriate content experts (typically the division chief or medical director of the involved area) to provide their input on the case. The goal is to complete the additional review in the next 24–48 hours. At the very latest, the event is to be determined by the next Event Review Meeting the following week.

### RCA—Time Reduction

We perceived the length of time from a determination that an RCA was needed (for either an SSE or a non-SSE with significant system issues) to the completion of the RCA and formation of an action plan as too long. A goal was set to reduce the average RCA completion time to <30 days, with 100% of cases completed in <30 days.

For the RCA process used at our children’s hospital, we adopted a two-meeting model from the three-meeting model described by HPI.^1^ This model was used for RCAs conducted during both the baseline and post-change periods. In the two-meeting model, we dedicate the first meeting to the review of the event timeline and identification of gaps in care. The second meeting is dedicated to the review of the identified system failures and the creation of countermeasures in the form of an action plan.

#### Baseline RCA Process.

In the baseline RCA process, patient safety advisors conducted and facilitated the meetings. The attendees consisted of representatives from the involved unit locations and service disciplines including management, front-line staff, providers, a quality manager, and subject matter experts. We included representatives from support departments such as accreditation regulatory and compliance, PPEC co-chairs, risk authority, family advisory council, and patient experience as needed. No owner of the RCA action plan, executive sponsor, or quality physician lead was determined.

Once we identified the participants in the RCA, an electronic scheduling poll was sent to determine a potential time for the members to meet. If this failed to yield a date that could accommodate a critical mass of members, additional polls with later dates were sent out until a date was determined. Once we agreed on a date, we scheduled a room for the meeting.

After we conducted the first RCA meeting, the patient safety advisors would draft the identified system failures for presentation at the second RCA meeting. During the second RCA meeting, the system failure modes were presented, edited, and a consensus established. Then the RCA team discussed potential countermeasures. After the second RCA meeting, the patient safety advisors would draft an action plan and send it out to the RCA members for approval and implementation. This process would usually take place 1–2 weeks following the second RCA meeting.

#### Causal Analysis for Factors Contributing to Lengthy RCA Process.

**Fig. 2.**
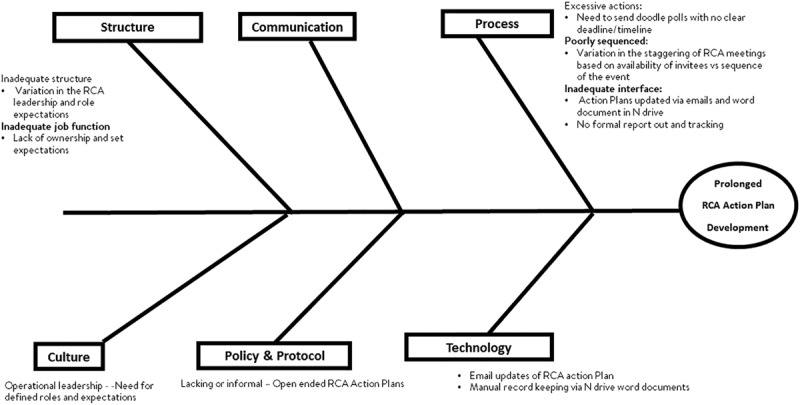
Cause and effect (Fishbone) diagram outlines factors which contributed to the long RCA completion times.

We performed a causal analysis to determine contributing factors to long determination times (Fig. 2). Dominant contributing factors included a cultural lack of emphasis by leadership to the urgency of the process, a scheduling process resulting in an inability to schedule the meeting, and a structural lack of clear roles and responsibilities among leadership to drive the process.

#### New RCA Process.

Most of the changes in the new process related to emphasizing the importance of timely completion of RCAs, the creation of action plans, the establishment of team membership, and assigning RCA leadership.

For each RCA, we assigned a member of the executive team, the chair of pediatrics, or the pediatric surgeon-in-chief as the RCA executive sponsor. This designation occurs at the weekly event review meetings upon determination of the need for an RCA. The executive sponsor is responsible for making sure the RCA meetings get scheduled in a timely fashion. The executive sponsor emphasizes the importance and priority of the RCA process, frees individuals for participation, and drives ownership for action plan creation and execution. An action plan owner is also assigned and is responsible for the creation and execution of the action plan. The owner can be a dyad and is most often the medical director and administrative director of the location or process most central to the incident. Also, there is a quality and safety physician assigned to the RCA to help with improvement methodology and action plan formation.

The two RCA meetings are scheduled to occur within a 30-day window starting from the day of determination of the need for an RCA. Commonly, RCA meetings are held between 12:00 and 2:00 pm with lunch provided. Time changes are possible to accommodate the team.

The patient safety advisors conduct and facilitate the RCA meetings. The attendees consist of representation from the involved locations and disciplines including management, front line staff, providers, a quality manager, and subject matter experts, as well as representation from support departments as needed, such as accreditation regulatory and compliance, PPEC co-chairs, risk authority, family advisory council, and patient experience. The goal is creating within 1 week after the second RCA meeting, an electronic copy of the action plan is distributed to all RCA members.

Once completed, the RCA action plan owner and executive sponsor present the plan to hospital executives at a standing monthly meeting. Presentations recur at 60-day intervals until the action plan is completed.

As the RCA process involves leaders from the pertinent PPECs, there typically are not both an RCA and a separate PPEC review. However, if the RCA leads to the discovery of other related professional issues, those identified issues can be brought or referred to the appropriate PPEC.

#### Data Analysis.

The mean times for SSE determination were compared using a two-sided *t* test assuming unequal variances for both cases that met the SSE criteria and were determined to be SSEs and cases that did not meet the SSE criteria. The mean times for RCA completion were compared using a two-sided *t* test assuming unequal variances. Statistical significance was defined as a *P*-value < 0.05. We performed statistical process control analysis with the use of Shewhart control charts. Control limits were calculated based on pre-intervention data. Based on these control limits, we looked for patterns in the post-intervention period that would permit us to invoke control chart rules that indicate a sustained decrease in the mean of the process. The evaluation was performed using Excel (Microsoft, Redmond, Wash.).

## RESULTS

**Fig. 3.**
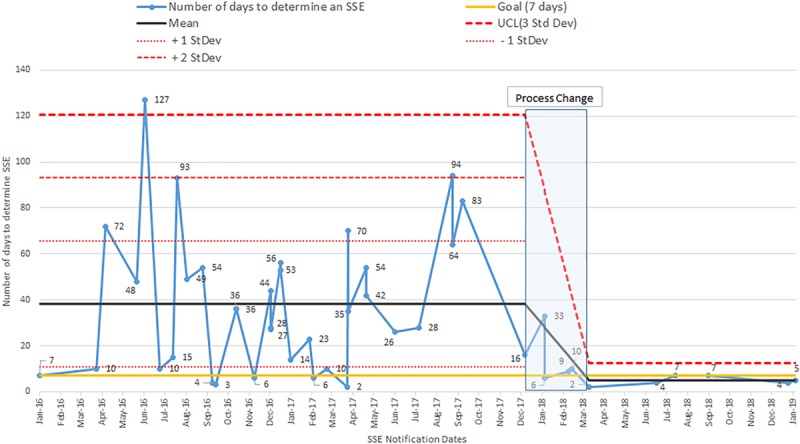
Individual SSE determination time for events meeting SSE criteria shown over time, comparing pre- vs post-implementation periods. Upper control limit (UCL) shown for three SDs.

We summarize the results in Table 1. The mean time for SSE determination for cases that met the SSE criteria for the pre-implementation phase was 38.4 ± 30.5 days, and for the post-implementation phase was 4.8 ± 1.9 days (Fig. 3, *P* < 0.0001). The number of SSEs called during the pre-implementation phase was 38, for the implementation phase was 4, and for the post-implementation phase was 6. The figure contains control limits based on the pre-intervention period. All 6 of the post-intervention times are <11, satisfying the Shewhart process change rule of 4 out of 5 points below 1 SD below the pre-intervention mean line.

**Table 1.**
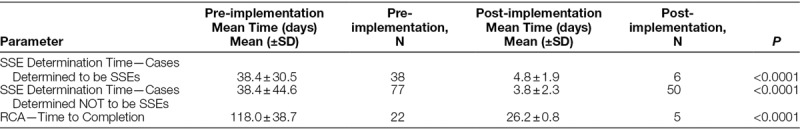
Comparison of SSE Determination Time and RCA Time to Completion Between Pre- and Post-Implementation Phases of Improvement Work

**Fig. 4.**
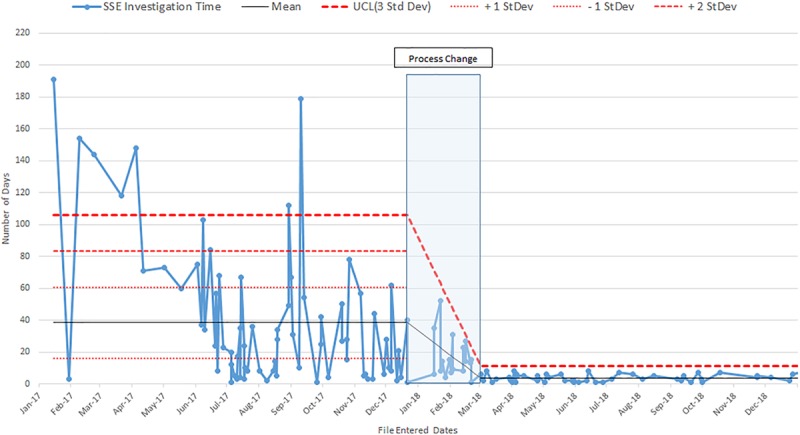
Individual determination time for events not determined to be SSEs shown over time, comparing pre- vs post-implementation periods. Upper control limit (UCL) shown for three SDs.

For cases brought to the SSE determination process that did not meet the SSE criteria, the mean time for determination for the pre-implementation phase was 38.4 ± 44.6 days and for the post-implementation phase was 3.8 ± 2.3 days (Fig. 4, *P* < 0.0001). The number of reviewed cases determined not to be SSEs during the pre-implementation phase was 77, for the implementation phase was 18, and for the post-implementation phase was 50. All of the post-intervention times are below one SD of the pre-intervention mean line, indicating a process change.

**Fig. 5.**
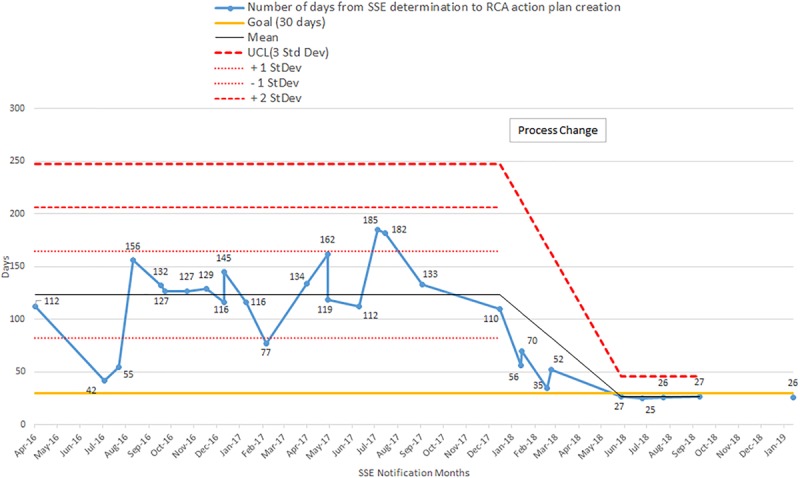
RCA completion times are shown, comparing pre- vs post-implementation periods. Upper control limit (UCL) shown for three SDs.

The mean time for RCA completion for the pre-implementation phase was 118.0 ± 38.7 days, and for the post-implementation phase was 26.2 ± 0.8 days (Fig. 5, *P* < 0.0001). During the post-implementation phase, we completed 100% of RCAs in <30 days. The number of RCAs conducted during the pre-implementation phase was 22, the implementation phase was 2, and the post-implementation phase was 5. All of the post-intervention times are below 1 SD of the pre-intervention mean line, indicating a process change.

## DISCUSSION

Numerous individual and groups of children’s hospitals have demonstrated that targeted programs to decrease SSEs can show a statistically significant positive effect on improving patient safety.^2–10^ The early adopting children’s hospitals worked with what was or would soon be HPI.^2–4^ Those successes were then spread via collaboratives; starting with the Ohio Children’s Hospitals Collaborative^5^ and followed by the SPS Collaborative.^6,7^ Due to this mechanism of spread and commonality of use of the HPI approach, most of the deployed programs were similar and focused on key drivers such as safety governance, error prevention training, reporting, and causal analysis, lessons learned the program, and specific tactical interventions for high-risk areas.^1–10^

Given the nature of SSEs, it is intuitive that timeliness in SSE determination, as well as RCA completion, is important in reducing the risk of recurrence of similar events. The impetus to decrease the time taken for SSE determination and RCA completion was an internally driven project, not related to an SPS initiative. The initiative to reduce SSE determination and RCA completion times was successful in that there was a statistically significant shortening of both time frames. We reduced SSE determination time by 7.6 times, from weeks to days, and RCA completion time by 4.5 times, from 4 to <1 month. The variability in how long it took to complete these tasks was also greatly reduced.

The key drivers to the reduction of SSE determination time were the implementation of a standardized process with defined roles of the participants, emphasis on the importance of timeliness by leadership, and rapid deployment of sub-subspecialty content experts when needed. By leadership emphasizing that timeliness of creating an action plan to prevent such occurrences from happening again was important helped create the culture that made these changes possible.

Numerous authors have written on the effectiveness of RCAs and the details on how they can be conducted.^1,11–15^ Our organization uses the two-meeting model based on the three-meeting mode used by SPS.^1–7^ As this was true both before and after the intervention, the key drivers to decreasing the length of time to execute the RCA are thought to be leadership emphasis on the importance of timeliness, the assignment of both an action plan owner and an executive sponsor, the structured content of the RCA, and the removal of scheduling impediments to conduct the RCA meetings.

A weakness of this study is that the new processes for both SSE determination and the RCA process were implemented in bundles. It is not clear which components of the process redesigns were the most important in improving the timeliness and which were noncontributory.

## CONCLUSION

In conclusion, this article shows that targeted intervention can significantly reduce SSE determination and RCA completion times. Other institutions, even those that have deployed SPS safety programs, may be able to benefit from a targeted initiative to reduce such times. By reducing these times, the vulnerability window during which a similar event may recur is shortened, and the likelihood of delivering high-reliability care increased.

## DISCLOSURE

The authors have no financial interest to declare in relation to the content of this article.
